# An Effective Vacuum Assisted Extraction Method for the Optimization of Labdane Diterpenoids from *Andrographis paniculata* by Response Surface Methodology

**DOI:** 10.3390/molecules20010430

**Published:** 2014-12-31

**Authors:** Ya-Qi Wang, Zhen-Feng Wu, Gang Ke, Ming Yang

**Affiliations:** Key Laboratory of Modern Preparation of Traditional Chinese Medicine, Ministry of Education, Jiangxi University of Traditional Chinese Medicine, Nanchang 330004, China; E-Mails: wangyaqi_3@163.com (Y.-Q.W.); zfwu527@163.com (Z.-F.W.); kgandrgl@gmail.com (G.K.)

**Keywords:** andrographolide, dehydroandrographolide, *Andrographis paniculata*, vacuum assisted extraction, response surface methodology (RSM)

## Abstract

An effective vacuum assisted extraction (VAE) technique was proposed for the first time and applied to extract bioactive components from *Andrographis paniculata*. The process was carefully optimized by response surface methodology (RSM). Under the optimized experimental conditions, the best results were obtained using a boiling temperature of 65 °C, 50% ethanol concentration, 16 min of extraction time, one extraction cycles and a 12:1 liquid-solid ratio. Compared with conventional ultrasonic assisted extraction and heat reflux extraction, the VAE technique gave shorter extraction times and remarkable higher extraction efficiency, which indicated that a certain degree of vacuum gave the solvent a better penetration of the solvent into the pores and between the matrix particles, and enhanced the process of mass transfer. The present results demonstrated that VAE is an efficient, simple and fast method for extracting bioactive components from *A. paniculata*, which shows great potential for becoming an alternative technique for industrial scale-up applications.

## 1. Introduction

**Figure 1:**
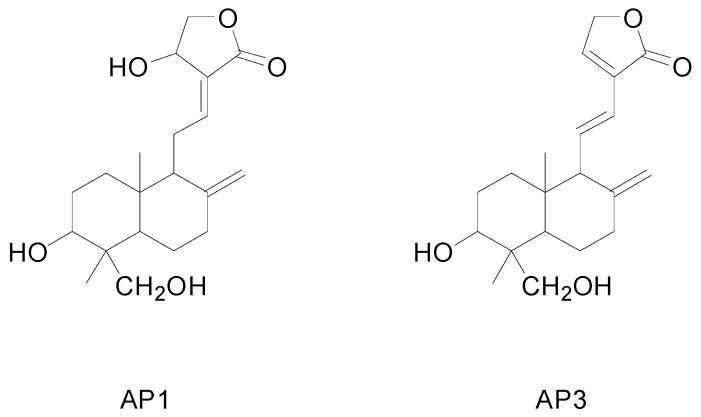
The chemical structure of andrographolide (AP_1_) and dehydroandrographolide (AP_3_).

*Andrographis paniculata* Nees, an annual herb in the Acanthaceae family, is one of the most famous herbal resources extensively used as a traditional medicine in China, India, Thailand, and Scandinavia for prevention and treatment of fever, dysentery, diarrhea, inflammation, sore throat, and snakebites [1,2]. Furthermore, it is a promising new way for the treatment of several serious diseases, including HIV [3], AIDS [4], and numerous symptoms associated with immune disorders [1,3]. The main active compounds in *A. paniculata* are believed to be the diterpenes, and andrographolide (AP_1_) and dehydroandrographolide (AP_3_, Figure 1) are the important and major diterpenoids in *A. paniculata* with a wide range of pharmacological activities, such as antiviral [3], anti-inflammatory [5], hepatoprotective [6], anticancer [7], immunostimulant [7], antiangiogenic [8] and antihyperglycemic [9].

Heat reflux extraction (HRE) is the most widely used conventional technique for the extraction of diterpenoids from *A. paniculata*. However, HRE causes the consumption of large amounts of volatile and hazardous organic solvents, and needs long extraction times and consumes more energy. Besides, the extraction efficiency of diterpenoids by conventional extraction method is not satisfactory [1,10]. Novel methods for the extraction of diterpenoids including supercritical carbon dioxide extraction [10], micellar extraction [11] and microwave assisted extraction [12] have drawn significant research attention in the past decade. However, complex equipment construction, high equipment expenditure and low material throughput have made them difficult in industrial scale-up applications [13]. Therefore, it is important to improve conventional extraction technique and establish an efficient, simple and fast extract method for industrial scale-up applications.

Vacuum assisted extraction (VAE) technology is developed based on pressurized liquid extraction (PLE) except in the vacuum controlled region [14]. A VAE device for the extraction of active compounds from traditional Chinese medicine has been designed in our research group [15]. It is a cheap, simple, fast and efficient method which can be implemented by simply upgrading the conventional equipment with the addition of a vacuum controller device. Like PLE, VAE can accelerate the release of solutes from the plant matrix by the vacuum assisted breakdown of cell components, and facilitate the solid-liquid mass transfer between the extraction solvent and matrix. The working pressure is stable and adjustable from 0 to 1000 mbar. In the VAE system, the extraction process is carried out at a temperature below the boiling point of the solvent by adjusting the vacuum setting of the system. The boiling temperature of the solvent is relative with the saturated vapor pressure above the solvents, so boiling at low temperatures can be realized by adjusting the system vacuum. Moreover, under vacuum, small bubbles appear and ascend among the liquid-solid phase, resulting in the violent movement of solvent and further improving the liquid-liquid mass transfer.

Nowadays, there is some literature describing vacuum-assisted extraction technologies, including vacuum microwave assisted extraction [16,17] and vacuum ultrasoound-assisted extraction [18], which could enhance the extraction efficiency and reduce the extraction time. Nevertheless, most of the studies are based on novel extraction techniques including ultrasonic-assisted and microwave-assisted extraction, which are difficult to use in industrial scaled-up applications due to the complex equipment construction [13]. HRE is still the most widely used conventional technique for the extraction of bioactive components from Chinese herbs in industry production processes. To our knowledge, there are no studies of vacuum-assist extraction methods based on the heat reflux extraction technique. The objective of this study was to explore the feasibility of VAE for extraction of diterpenoids from *A. paniculata*. The effects of boiling temperature, ethanol concentration, extraction time, extraction cycles, and ratio of liquid to solid were investigated. Response surface methodology (RSM) was used to build a model between the yield value and these independent variables, and to optimize the extraction conditions of diterpenoids from *A. paniculata* in order to provide valuable information for industrial purposes.

## 2. Results and Discussion

### 2.1. Actualization of the Vacuum Assisted Heat Reflux Extraction

**Figure 2:**
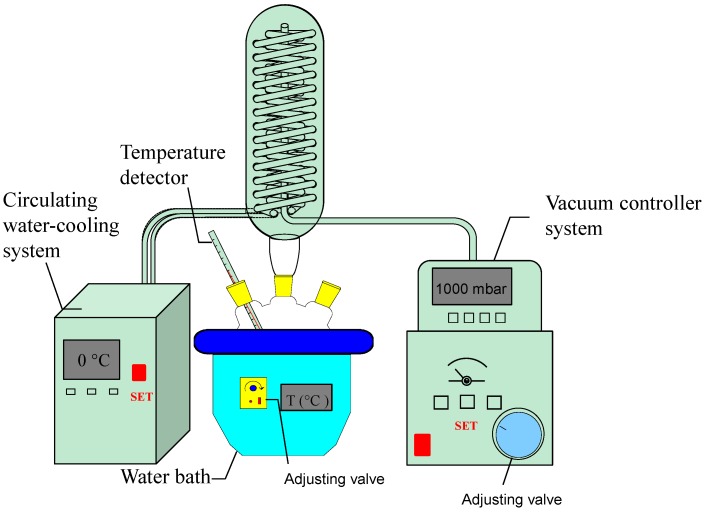
The VAE apparatus.

VAE has been performed with the apparatus shown in Figure 2. It is equipped with a vacuum controller, reflux heating device and a circulating water-cooling system. In this study, the vacuum controller was used to adjust the system vacuum. The working pressure was thus stable and adjustable from 0 to 1000 mbar. The boiling point of the solvent is related to the saturated vapor pressure above the solvents, so boiling at low temperature could be realized by adjusting the system vacuum. The boiling temperature decreased with the increase of vacuum. The relationship between the vacuum and boiling temperature of the solvent is shown in Table 1.

**Table 1:** Relationship among the vacuum, ethanol concentration and boiling temperature.

Ethanol Concentration	Boiling Temperature							
	50 °C		60 °C		70 °C		80 °C	
	Saturated Vapor Pressure (MPa)	Vacuum (MPa)	Saturated Vapor Pressure (MPa)	Vacuum (MPa)	Saturated Vapor Pressure (MPa)	Vacuum (MPa)	Saturated Vapor Pressure (MPa)	Vacuum (MPa)
50%	0.0231	0.0769	0.0366	0.0634	0.0571	0.0429	0.0847	0.0153
60%	0.0243	0.0757	0.0389	0.0611	0.0607	0.0393	0.0894	0.0106
70%	0.0248	0.0752	0.0407	0.0593	0.0636	0.0364	0.0935	0.0065
80%	0.0247	0.0753	0.0417	0.0583	0.0659	0.0341	0.0970	0.0030
90%	0.0239	0.0761	0.0422	0.0578	0.0676	0.0324	0.0999	0.0001

### 2.2. Key Factors Screening through Placket-Burman Design

A Placket-Burman design was used to screen variables in experiments resulting in a tremendous decrease in their total number. In order to screen the important variables affecting the diterpenoids production from *A. paniculata*, influences of five variables (including boiling temperature, ethanol concentration, extraction time, extraction cycles and ratio of liquid to solid) on extraction efficiency were investigated.

Each variable was tested at two levels of concentrations, namely a high level denoted by (+1) and a low level denoted by (−1), as listed in Table 2. ANOVA analysis provided the weights of the experimental variables for all of the responses. The *p*-values were used as instruments to check the significance of each coefficient. A small *p*-value would indicate a more significant effect on the corresponding coefficient. Test for significance of regression coefficients were shown in Table 2.

For the extraction yield of AP_1_ and AP_3_: the model *F*-value of 32.77 and 31.11, and the *p*-value were both 0.0003, which implied that the two model were significant and indicated that the two fitted models were suitable for use in this experiment. *P*-value less than 0.05 indicated model terms were significant. In these cases, boiling temperature (A) and ethanol concentration (B) exhibited great significant (*p* < 0.01) effect on the extraction efficiency of the two active components. On the other hand, the extraction time (C), extraction cycles (D) and ratio of liquid to solid (E) showed little effect (*p* > 0.05) on the yield value of AP_1_ and AP_3_. For ES, the model *F*-value of 19.90 and the *p*-value were 0.0011, which implied that the model was significant and indicated that the model was suitable for use in this experiment. In these cases, boiling temperature (A), ethanol concentration (B), extraction cycles (D) exhibited significant (*p* < 0.05) effect on the yield. The sequence of main variables respect to decreasing of influence on yield value of ES was B > D > A > C > E. Table 2 showed that the yield value of AP_1_ and AP_3_ couldn’t rise with the extraction cycles. Therefore, from the economic perspective, we fixed the extraction cycles (D) at 1 and the ratio of liquid to solid (E) at 12 in this study, and the other variables including boiling temperature (A), ethanol concentration (B) and extraction time (C) were chosen to further optimized by the subsequent Box-Behnken design.

**Table 2:** Arrangement and results of the Placket-Burman design.

Run	Factors					Response Variables		
	A (Boiling Temperature)	B (Ethanol Concentration)	C (Extraction Time)	D (Extraction Cycles)	E (Ratio of Liquid to Solid)	AP_1_ (%)	AP_3_ (%)	ES (%)
1	80 (+1)	90 (+1)	40 (+1)	1 (−1)	12 (+1)	81.40	84.71	5.82
2	40 (−1)	90 (+1)	10 (−1)	1 (−1)	6 (−1)	58.24	52.37	3.84
3	80 (+1)	90 (+1)	40 (+1)	3 (+1)	6 (−1)	75.41	70.77	7.82
4	40 (−1)	90 (+1)	40 (+1)	1 (−1)	6 (−1)	52.42	47.89	3.96
5	40 (−1)	50 (−1)	40 (+1)	3 (+1)	12 (+1)	58.70	55.51	8.78
6	80 (+1)	50 (−1)	40 (+1)	1 (−1)	12 (+1)	107.48	105.83	8.36
7	80 (+1)	50 (−1)	10 (−1)	3 (+1)	6 (−1)	107.22	106.07	9.44
8	40 (−1)	90 (−1)	10 (−1)	3 (+1)	12 (+1)	52.24	45.37	5.54
9	40 (−1)	50 (−1)	10 (−1)	1 (−1)	12 (+1)	62.08	55.87	6.42
10	40 (−1)	50 (−1)	40 (+1)	3 (+1)	6 (−1)	58.71	55.51	8.78
11	80 (+1)	50 (−1)	10 (−1)	1 (−1)	6 (−1)	113.22	108.07	8.44
12	80 (+1)	90 (+1)	10 (−1)	3 (+1)	12 (+1)	89.58	85.13	5.14
		AP_1_ (%)		AP_3_ (%)		ES (%)		
	Source	*F*-value	*p*-value	*F*-value	*p*-value	*F*-value	*p*-value	
	Model	32.77	0.0003	31.11	0.0003	19.90	0.0011	
	A (boiling temperature)	131.44	0.0001	129.12	0.0001	12.04	0.0133	
	B (ethanol concentration)	23.53	0.0029	21.25	0.0037	66.68	0.0002	
	C (extraction time)	5.74	0.0535	2.24	0.1851	4.50	0.0782	
	D (extraction cycles)	2.66	0.1539	2.78	0.1467	15.29	0.0079	
	E (ratio of liquid to solid)	0.46	0.5229	0.14	0.7178	1.00	0.3568	

### 2.3. Optimization of Screened Variables for the Diterpenoids Production Using Response Surface Methodology

Three influential variables of the VAE procedure were screened for further investigations based on Box-Behnken design. Table 3 shows the 17 experimental runs with different combinations of the three variables along with experimental responses. All the experimental data was fitted to the quadratic model by ANOVA. Test for significance of regression coefficients is shown in Table 4.

**Table 3:** Arrangement and results of the Box-Behnken design.

Run	Factors			Response Variables		
	A (Boiling Temperature)	B (Ethanol Concentration)	C (Extraction Time)	AP_1_ (%)	AP_3_ (%)	ES (%)
1	40	50	25	95.20	93.94	7.39
2	80	70	10	125.89	111.12	8.14
3	80	70	40	109.07	106.97	8.94
4	80	90	25	91.81	85.2	4.95
5	40	90	25	59.85	56.39	1.79
6	60	70	25	96.45	92.53	6.25
7	60	70	25	90.52	85.59	6.87
8	60	90	10	75.40	72.48	2.62
9	80	50	25	148.37	132.37	9.34
10	60	50	10	136.76	120.42	7.99
11	60	70	25	109.92	106.27	5.74
12	40	70	40	65.77	74.03	5.85
13	60	90	40	65.89	67.94	3.62
14	40	70	10	65.03	66.27	4.50
15	60	50	40	129.69	127.99	9.24
16	60	70	25	103.18	100.97	6.10
17	60	70	25	98.39	95.32	5.62

**Table 4:** Analysis of variance (ANOVA) for the regression models.

Response	Source	Sum of Square	D.f.	Mean Square	*F*-value	*p-*value	
AP_1_ (%)	Model	10944.05	9	1216.01	21.46	0.0003	significant
	A	4478.84	1	4478.84	79.05	0.0001	
	B	5889.92	1	5889.92	103.96	0.0001	
	C	133.33	1	133.33	2.35	0.1689	
	AB	112.47	1	112.47	1.99	0.2017	
	AC	77.09	1	77.09	1.36	0.2816	
	BC	1.49	1	1.49	0.026	0.8758	
	A^2^	136.31	1	136.31	2.41	0.1648	
	B^2^	97.22	1	97.22	1.72	0.2316	
	C^2^	27.64	1	27.64	0.49	0.5074	
	Residual	396.6	7	56.66			
	Lack of fit	183.49	3	61.16	1.15	0.4316	not significant
	Pure error	213.11	4	53.28			
	Total	11340.65	16				
AP_3_ (%)	Model	7529.74	9	836.64	17.61	0.0005	significant
	A	2629.61	1	2629.61	55.34	0.0001	
	B	4642.14	1	4642.14	97.70	0.0001	
	C	5.51	1	5.51	0.12	0.7434	
	AB	23.14	1	23.14	0.49	0.5078	
	AC	35.46	1	35.46	0.75	0.4162	
	BC	36.66	1	36.66	0.77	0.4088	
	A^2^	145.85	1	145.85	3.07	0.1232	
	B^2^	12.52	1	12.52	0.26	0.6235	
	C^2^	1.80	1	1.80	0.038	0.8514	
	Residual	332.59	7	47.51			
	Lack of fit	81.64	3	27.21	0.43	0.7407	not significant
	Pure error	250.95	4	62.74			
	Total	7862.33	16				
ES (%)	Model	78.06	9	8.67	42.50	0.0001	significant
	A	17.52	1	17.52	85.87	0.0001	
	B	55.02	1	55.02	269.63	0.0001	
	C	2.42	1	2.42	11.86	0.0108	
	AB	0.37	1	0.37	1.79	0.2223	
	AC	0.076	1	0.076	0.37	0.5619	
	BC	0.016	1	0.016	0.077	0.7900	
	A^2^	0.58	1	0.58	2.84	0.1360	
	B^2^	1.61	1	1.61	7.91	0.0260	
	C^2^	0.58	1	0.58	2.84	0.1360	
	Residual	1.43	7	0.20			
	Lack of fit	0.45	3	0.15	0.62	0.6372	not significant
	Pure error	0.97	4	0.24			
	Total	79.49	16				

#### 2.3.1. Response Surface of the Extraction Yield of AP_1_ and AP_3_

For the extraction yield of AP_1_ and AP_3_: the model *F*-value of 21.46 and 17.61, and the *p*-value were 0.0003 and 0.0005, which implied that the two model were significant and there were only a 0.03% and 0.05% chance that a “Model *F*-value” this large could occur due to noise, which indicated that the two fitted models were suitable for use in this experiment. *P*-value less than 0.05 indicated model terms were significant. In these cases, Table 4 indicated the effect of the A (boiling temperature) and B (ethanol concentration) were significant model terms with a negative linear relationship (*p* < 0.01) on both responses, whereas the effect of C (extraction time) was not significant (*p* > 0.05). The “Lack of Fit *F*-value” of 1.15 and 0.43 implied the Lack of Fit were not significant relative to the pure error. There were a 43.16% and 74.07% chance that a “Lack of Fit *F*-value” this large could occur due to noise. Non-significant lack of fit was good—we wanted the model to fit, which further validated the model. The polynomial models of the extraction yield of AP_1_ and AP_3_ were regressed according to the designed experimental data and presented in Equations (1) and (2) in term of coded factors:

(1) $$\begin{array}{c} AP_{1}\;(\%)=99.69+23.66A-27.13B-4.08C-5.30AB-4.39AC-0.61BC-\\ 5.69A^{2}+4.81B^{2}-2.56C^{2} \end{array}$$

(2) $$\begin{array}{c} AP_{3}\;(\%)=96.14+18.13A-24.09B+0.83C-2.41AB-2.98AC-3.03BC-\\ 5.89A^{2}+1.72B^{2}-0.65C^{2} \end{array}$$

**Figure 3:**
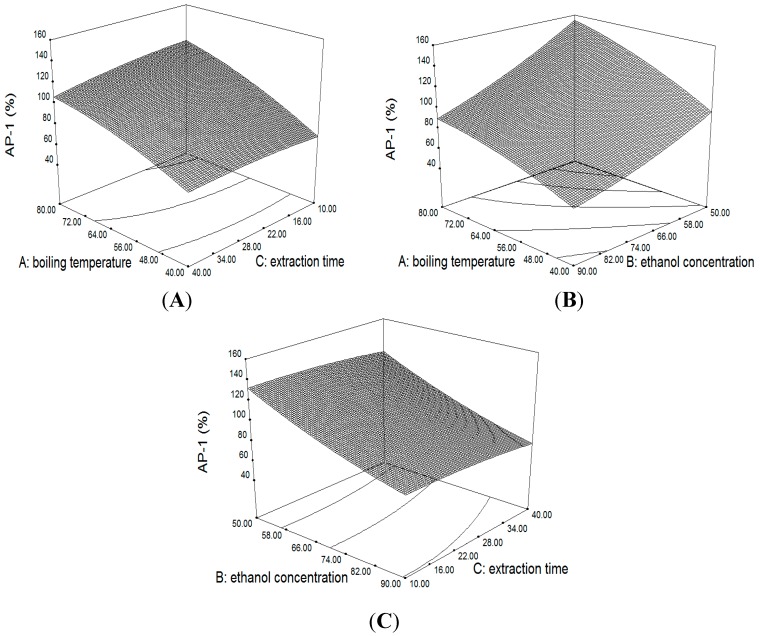
3D surface and contour plot showing the effect of variables on the extraction yield of AP_1_.

The value for the coefficient of determination (*R*^2^) for extraction yield of AP_1_ and AP_3_ were 0.9650 and 0.9577, respectively, which implied that over 96.5% and 95.77% of the variations for the process efficiency could be explained by these models. The closer *R*^2^ to 1, the better the empirical models fits the actual data. To consider the interaction of different extraction parameters, the three-dimensional surface and contour plot of multiple non-linear regression models were depicted in Figure 2 and Figure 3.

**Figure 4:**
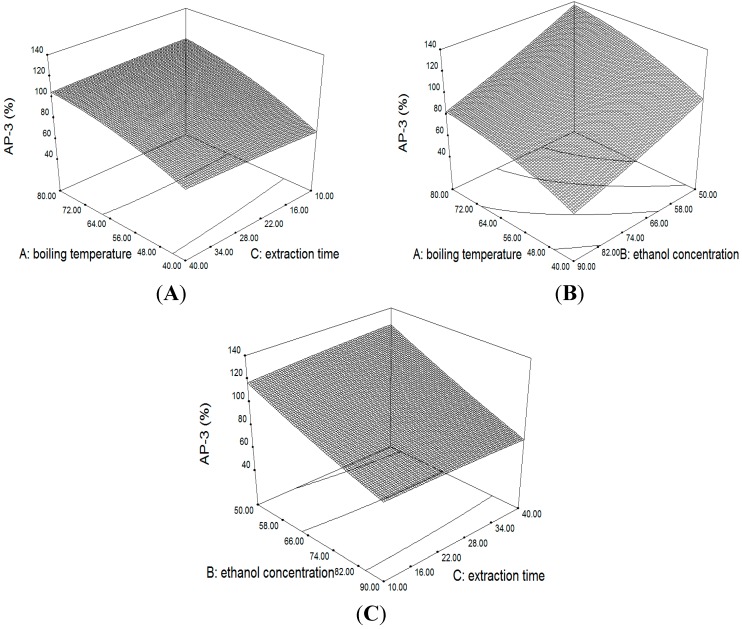
3D surface and contour plot showing the effect of variables on the extraction yield of AP_3_.

Boiling temperature had a significant positive effect on the extraction efficiency of AP_1_ and AP_3_ (*p* < 0.001). The results clearly showed that the AP_1_ and AP_3_ yield was increased with boiling temperature (Figure 3A,B and Figure 4A,B). This phenomenon may be caused by the low rate of mass transfer at low temperatures (high degree of vacuum), which would require more time to dissolve compounds from the plant materials into the solution. At higher temperatures, dissolution of the analytes can reach the equilibrium in a shorter time thus are not readily affected by changes in the extraction time [19]. This also indicated that a lower vacuum and a short extraction time are more effective in extracting diterpenoids from *A. paniculata* by VAE method. Besides, several authors proved that prolonged exposure to high temperature will accelerate the thermal degradation of diterpenoids in *A. paniculata* [20,21,22].

Ethanol concentration had a significant negative effect on the extraction efficiency of AP_1_ and AP_3_ (*p* < 0.001). The results showed that the yield value decreased dramatically as the ethanol concentration increased from 50% to 90% (Figure 3B,C and Figure 4B,C). There was a 61% and 47% decrease in extraction efficiency of AP_1_ and AP_3_ from 50% to 90%. *A. paniculata* included a large variety of chemical components such as diterpenoids, glycosides, flavones and sterols. Thereinto, glycosides have relatively high affinity with water due to their polarity. Therefore, the possible reasons is that 50% ethanol as extraction solvent could enhance the solubility of diterpene glycosides, and then improve the mass transfer of target diterpenoids due to the solubilization of glycoside. A similar result has been reported by Zhang [23,24].

Figure 3B and Figure 4B showed the interactive effects of boiling temperature and ethanol concentration. When the ethanol concentration were at 50%–90%, the value of AP_1_ and AP_3_ increased as the boiling temperature went up, but was not significantly changed by the ethanol concentration. ANOVA analysis (Table 4) also showed that the interactive effect between boiling temperature and ethanol concentration on the yield value of AP_1_ and AP_3_ were insignificant (*p* > 0.05).

#### 2.3.2. Response Surface of the Yield of ES

For the yield of ES: The Model *F*-value of 42.5 implied the model was significant. There was only a 0.01% chance that a “Model *F*-Value” this large could occur due to noise. A, B, C and B^2^ were significant model terms, where *p*-value less than 0.05. The “Lack of Fit *F*-value” of 0.62 implied the Lack of Fit was not significant relative to the pure error. There was a 63.72% chance that a “Lack of Fit *F*-value” this large could occur due to noise. The “Pred *R*^2^” of 0.8894 was in reasonable agreement with the “Adj *R*^2^” of 0.9589. “Adeq Precision” showed 23.682 and indicated this model can be used to navigate the design space.

Similarly, the final predictive empirical model of the yield of ES was regressed according to the designed experimental data and presented in Equation (3) in term of coded factors:

(3) $$\begin{array}{c} ES\;(\%)=6.12+1.48A-2.62B+0.55C+0.30AB-0.14AC-0.063BC+\\ 0.37A^{2}-0.62B^{2}+0.37C^{2} \end{array}$$

As evident in Figure 4 and Table 4, the A (boiling temperature) and C (extraction time) had significant positive effects, whereas the effect of B (ethanol concentration) on the ES yield was also significant with a negative linear relationship (*p* < 0.05).

**Figure 5:**
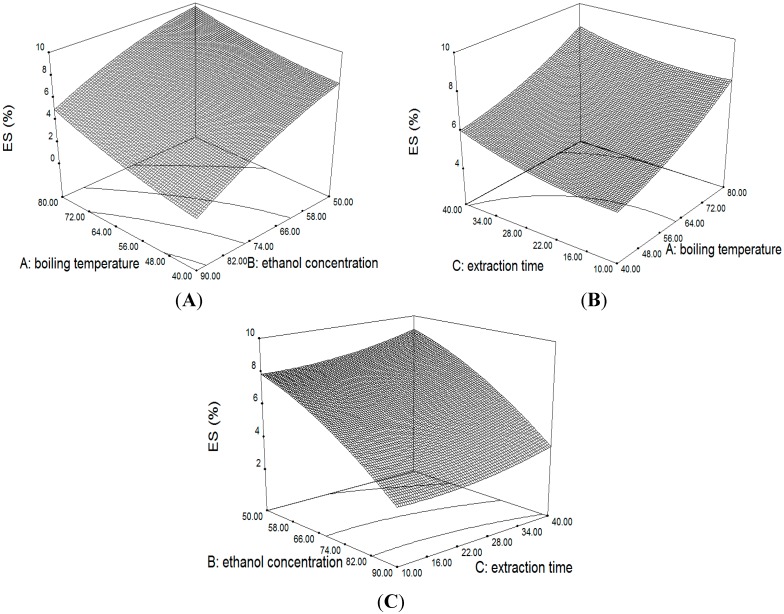
3D surface and contour plot showing the effect of variables on the yield of ES.

In general, the relationship of ES was similar to what was observed in AP_1_ and AP_3_. At a fixed boiling temperature of 80 °C and extraction time of 25 min, an increase in the ethanol concentration from 50% to 90% caused a 4.39% decrease in ES% (Figure 5A), fixed boiling temperature of 80 °C and ethanol concentration of 70%, the increase in extraction time from 10 to 40 min led to a 0.8% increase in ES% (Figure 5B), and fixed ethanol concentration of 50% and extraction time of 25 min, the increase in boiling temperature of 40 °C to 80 °C led to a 1.95% increase in ES% (Figure 5C).

#### 2.3.3. Optimization of the Extraction Process

In this study, the purpose of optimization was to obtain the conditions which could provide the maximum extraction yield of diterpenoids and minimum *extracta sicca* yield. A desirability function approach was used to achieve this goal of optimizing the three responses simultaneously. Each response can be assigned a significance degree relative to the other responses. Table 5 showed the constraints which were set in the software for the optimization. The final optimum extraction conditions were: boiling temperature, 65 °C, extraction time, 16 min, ethanol concentration, 50%.

**Table 5:** Responses for optimization and verification experiments.

Response	Goal	Importance	Replicate 1	Replicate 2	Replicate 3	Predicted Value
AP_1_ (%)	Maximize	5	135.3	139.6	138.3	138.3
AP_3_ (%)	Maximize	5	120.4	123.3	122.6	123.0
ES (%)	Minimize	3	7.9	8.0	8.1	8.12

### 2.4. Verification

Table 5 shows the results of three parallel experiments which were run at the optimized parameters to validate the final predictive empirical models. The mean value of extraction yield of AP_1_ and AP_3_ were 137.7% and 122.1%, and the average of yield of ES was 8.0% in the checking experiments. Comparing with the values predicted by Design-Expert 8.0.6 (Table 5), the actual results showed very close agreement. The good correlation between these results indicated that the response models were reliable in predicting the optimized conditions.

### 2.5. Comparison

Comparison experiments were performed in order to better clarify the advantages and disadvantages of VAE. The reference methods include HRE (RE1), UAE (RE2) and modified HRE (RE3). Table 6 summarizes the results of comparative studies on the three responses obtained by VAE and conventional extraction methods including HRE (RE1), UAE (RE2) and modified HRE (RE3). Apparently, the optimized VAE gave the best extraction efficiency compared to the other three reference methods. With a comparison between VAE and RE3, under the same extraction conditions, VAE showed a significant improvement of extraction efficiency of AP_1_ and AP_3_, which indicated that a certain degree of vacuum can accelerate the pace of penetration of the solvent into the pores and between the matrix particles, and enhances the process of mass transfer [24,25]. The UAE has the similar advantages in extraction yield of AP_1_ and AP_3_, but a higher yield of ES and longer extraction time as compared with VAE. Therefore, it was clear that applying VAE can significantly improve extraction efficiency while reducing extraction time, and had a great potential to be a rapid and effective approach for extraction of diterpenoids from *A. paniculata*.

**Table 6:** Comparison experiments.

Extraction Method	Ethanol Concentration (%)	Extraction Time (min)	AP_1_ (%)	AP_3_ (%)	ES (%)
RE1	85	240	100.00	100.00	10.0
RE2	40	30	129.26	120.74	16.5
RE3	50	16	89.63	92.11	9.0
VAE	50	16	137.7	122.1	8.0

## 3. Experimental Section

### 3.1. Materials

*A. paniculata* was obtained from a local drugstore in Nanchang (China). Reference samples of andrographolide (AP_1_) and dehydroandrographolide (AP_3_) were supplied by the National Institute for the Control of Pharmaceuticals and Biological Products (Beijing, China). Ethanol (analytical grade), acetonitrile (chromatographic grade), and formic acid (chromatographic grade) were obtained from local chemical suppliers.

### 3.2. Apparatus

An Agilent 1200 HPLC system equipped with a quaternary solvent delivery system, an autosampler and variable-wavelength ultraviolet detector (VWD) (Agilent Technologies, Santa Clara, CA, USA) was used for HPLC analysis. A Phenomenex reversed-phase Gemini C_18_ column (250 × 4.6 mm, 5 μm) and a Phenomenex C_18_ guard column (Phenomenex, Torrance, CA, USA) were used for all chromatographic analysis. VAE experiments were carried out with a V850 Vacuum Controller (Büchi Labortechnik AG, Flawil, Switzerland).

### 3.3. High Performance Liquid Chromatograph Analysis

**Figure 6:**
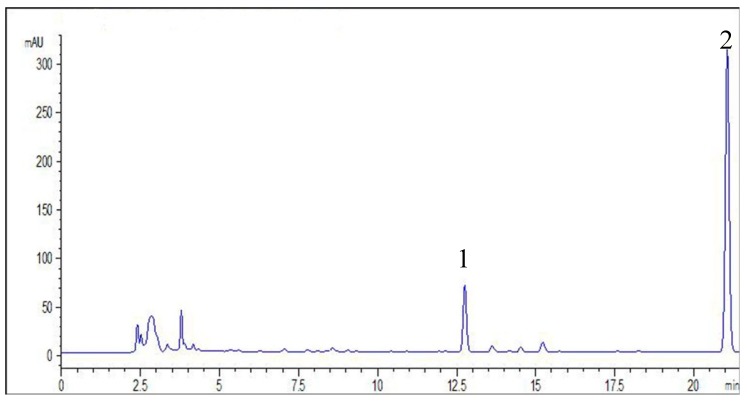
HPLC chromatograms of diterpenoids from *A. paniculata*. Peak 1, andrographolide (AP_1_) and peak 2, dehydroandrographolide (AP_3_)

Determination of diterpenoids extracted from *A. paniculata* by various extraction methods was analyzed by the HPLC method. The detection wavelength was 254 nm. The gradient elution system consisted of acetonitrile (solvent A) and water with 0.2% formic acid (solvent B). Separation was achieved using the following gradient procedures: 0–20 min, 25%A–50%A; 20–25 min, 50%A–100%A. The flow rate of mobile phase was set at 1.0 mL/min, the injection volume was 10 μL, and the column temperature was maintained at 30 °C. The linear range of AP_1_ and AP_3_ were 38.4–192 μg/mL (R = 0.9996), 16–80 μg/mL (R = 0.9996), respectively. The results implied the HPLC method was reliable for quantitative analysis of AP_1_ and AP_3_ (Figure 6).

### 3.4. Vacuum Assisted Extraction (VAE)

*A. paniculata* (10 g) was soaked for 1 h with three times the volume of extraction solvent. Extraction was carried on according to the experimental design. The extraction process was performed using a vacuum controller, with different vacuum degree and extraction time settings. After being extracted, the mixture was filtered under vacuum through Whatman No.1 paper (Whatman-Xinhua Filter Paper Co., Zhejiang, China). The extracts were united HPLC analysis and weight measurement of the extracta sicca. The extraction efficiency of AP_1_ and AP_3_ was calculated according to Equation (4). The yield of extracta sicca (ES) was calculated according to Equation (5). The quantity of andrographolide and dehydroandrographolide in HRE extract was 4.05 mg/g (w/w) and 2.25 mg/g (w/w), respectively. The determination methods for the values were from Chinese Pharmacopoeia [26]: *A. paniculata* was soaked with ethanol-water (85:15, v/v) for 1 h and reflux-extracted for 2 times, 2 h every time, then the extracting solution is merged and analyzed by HPLC:

(4) $$\text{Extraction efficiency (\%)=}\frac{\text{quantity of analyte in VAE extract }}{\text{quantity of analyte in HRE extract}}\;\times \text{ 100}$$

(5) $$\text{Yield of ES (\%)}=\frac{\text{mass of extracta sicca}}{\text{mass of plant material }}\;\times \;100$$

### 3.5. Response Surface Methodology (RSM)

RSM was applied in two stages, first to identify the significant factor for extraction efficiency of AP_1_ and AP_3_ and yield of *extracta sicca* using a Placket-Burman design and later the significant variables resulted from the Placket-Burman design were optimized by using a Box-Behnken design. All experiments aimed at optimizing the conditions of extraction for greatest efficiency. Each experiment was conducted in triplicate and the average yield value of AP_1_, AP_3_ and ES was used for statistical analysis.

The experiments were carried out in random order to avoid systematic errors. The statistical software package Design-expert 8.0.6 was used for regression analysis of the data and estimation of the regression equation coefficients. A second-order response function was applied to establish an empirical model that relates the response measured to the independent variables. This is shown in Equation (6):

(6) $$Y\;=b_{0}+\sum_{i=1}b_{i}X_{i}+\sum_{i=1}b_{ii}X_{i}^{2}+\sum_{i,j=1}b_{ij}X_{ij}$$

where *Y* is the measured response variable, *b*_0_ is a constant, *b_i_* is the linear coefficient, *b_ii_* is the quadratic coefficient, *b_ij_* is the two factors interaction coefficient, and *X_i_* and *X_j_* are independent variables of the system. The quality of the fitted polynomial model was expressed by the coefficient of determination (*R*^2^), and its statistical significance was checked by *p*-test and *F*-test.

### 3.6. Optimization

A desirability function approach was used to optimize the three responses simultaneously. Each response can be assigned a significance degree relative to the other responses:

(7) $$D={\left(d_{1}r_{1}\times d_{2}r_{2}\times \ldots \times d_{n}r_{n}\right)}^{\frac{1}{\sum r_{i}}}={\left(\prod_{i\text{=}1}^{n}d_{i}r_{i}\right)}^{\frac{1}{\sum r_{i}}}$$

where *d_i_* is the partial desirability function of each response obtained from the transformation of the individual response of each experiment. *r_i_* is the significance degree of each response.

### 3.7. Reference Extraction Methods

In the Chinese Pharmacopoeia, heat reflux extraction (HRE) is the traditional extraction process of Andrographis tablets. Thus the first reference extraction method (RE1) was operated using 85% ethanol aqueous solution as extraction solvent and two reflux extractions for 2 h every time. The second reference extraction method (RE2) was conducted with ultrasound assistance (UAE) by using 40% ethanol aqueous solution for 30 min, after immersing in the same solvent for 1 h beforehand. The third reference extraction method (RE3) was conducted by using 50% ethanol aqueous solution as extraction solvent for 16 min under the optimized VAE conditions, except for the vacuum assistance (modified HRE).

## 4. Conclusions

In this study, an effective vacuum assisted extraction method was successfully used to extract diterpenoids from *A. paniculata*. RSM with a Placket-Burman design, Box-Behnken design and desirability function were employed to get the optimal extraction conditions for diterpenoids in a quick and economical way. Coefficient of determination of the three models suggested good fit. Compared with conventional HRE, bioactive components can be efficiently extracted from *A. paniculata* by the optimal VAE conditions (boiling temperature 65 °C, ethanol concentration 50%, extraction time 16 min, extraction cycles 1 and liquid-solid ratio 12:1) and the extraction yields of AP_1_ and AP_3_ was 137.7% and 122.1%, the yield of ES was 8.0%. The experimental results proved a good accordance with the predicted values. The optimized VAE not only accelerated the extraction rate and improved the efficiency of the extraction yield of bioactive components, but also shortened the extraction time and energy compared to conventional HRE, which shows great potential for becoming an alternative technique for industrial scale-up applications.
